# Using Advanced Convolutional Neural Network Approaches to Reveal Patient Age, Gender, and Weight Based on Tongue Images

**DOI:** 10.1155/2024/5551209

**Published:** 2024-08-01

**Authors:** Xiaoyan Li, Li Li, Jing Wei, Pengwei Zhang, Volodymyr Turchenko, Naresh Vempala, Evgueni Kabakov, Faisal Habib, Arvind Gupta, Huaxiong Huang, Kang Lee

**Affiliations:** ^1^ Hangzhou Normal University Affiliated Hospital, Hangzhou, Zhejiang, China; ^2^ Computer Science University of Toronto, Toronto, Ontario, Canada; ^3^ Nuralogix Corp., Toronto, Ontario, Canada; ^4^ Mathematics, Analytics, and Data Science Lab Fields Institute for Research in Mathematical Sciences, Toronto, Ontario, Canada; ^5^ Mathematics and Statistics York University, Toronto, Ontario, Canada

## Abstract

The human tongue has been long believed to be a window to provide important insights into a patient's health in medicine. The present study introduced a novel approach to predict patient age, gender, and weight inferences based on tongue images using pretrained deep convolutional neural networks (CNNs). Our results demonstrated that the deep CNN models (e.g., ResNeXt) trained on dorsal tongue images produced excellent results for age prediction with a Pearson correlation coefficient of 0.71 and a mean absolute error (MAE) of 8.5 years. We also obtained an excellent classification of gender, with a mean accuracy of 80% and an AUC (area under the receiver operating characteristic curve) of 88%. ResNeXt model also obtained a moderate level of accuracy for weight prediction, with a Pearson correlation coefficient of 0.39 and a MAE of 9.06 kg. These findings support our hypothesis that the human tongue contains crucial information about a patient. This study demonstrated the feasibility of using the pretrained deep CNNs along with a large tongue image dataset to develop computational models to predict patient medical conditions for noninvasive, convenient, and inexpensive patient health monitoring and diagnosis.

## 1. Introduction

The human tongue is a vital organ with many important functions. Within traditional Chinese medicine, the tongue has been referred to as the magnifying glass of human health because it has close connections to various organs and physiological activities. Thus, it has been long believed that the tongue can be used to provide important insights into a patient's health [1–6]. Physicians often examine the tongue during a patient visit, both in western and eastern medicine. Despite the tongue's importance in clinical diagnosis, there has been limited scientific research on how the tongue can be used to provide accurate information about a patient's health conditions. As an initial step to address this gap in the literature, the present research is aimed at exploring the use of advanced machine learning techniques to accurately predict patients' age, gender, and weight based on the photographs of the tongue.

The human tongue is a muscular organ located in the oral cavity and is responsible for several functions, including tasting, swallowing, and speaking. The tongue is made up of several different types of muscles, including intrinsic and extrinsic muscles, which have rich vasculatures. The tongue is covered by a thin layer of the mucous membrane, which is lined with tiny bumps called papillae. These papillae contain taste buds, which are responsible for detecting different flavors. The tongue is also richly innervated, which allows it to detect different textures and temperatures. In addition to its role in taste and speech, the tongue also plays a role in digestion by moving food or liquid around in the mouth, mixing it with saliva, and preparing it to be swallowed. Saliva contains enzymes that begin the process of breaking down food, and the tongue helps to mix and distribute these enzymes throughout the food as it is being chewed. Overall, the tongue is a complex and multifunctional organ that is essential for many physiological processes.

Given its multiple functions and close connections to many different systems in the body, the tongue has long been hypothesized to be a valuable organ for identifying potential health issues. Indeed, the physical characteristics of the tongue, such as its color, shape, sores, or bumps, can serve as indicators of underlying health conditions [7–11]. For this reason, the doctors practicing traditional Chinese medicine have long relied on examining the tongue to diagnose illnesses and prescribe treatments [4, 12]. These guidelines involve observing the color variations, coating, shape, moisture, and other features of the tongue. In recent years, western medicine has also increasingly embraced this practice, with some doctors incorporating tongue examination, adhering to similar guidelines for its outer manifestations into their clinical protocol [13–16]. Despite the clinical importance of the tongue, scientific understanding of the tongue's role in diagnosis has been lacking.

The present study seeks to explore whether the use of machine learning algorithms along with tongue images is able to accurately predict a patient's age, gender, and weight as a proof-of-concept. We focused on the prediction of patient age, gender, and weight because these measurements may consist relevant information of a patient. Age is a major risk factor for various diseases [17, 18]. Gender is also strongly associated with different diseases and can play a role in the diagnosis and treatment of a patient [19–21]. Weight is also closely associated with health and is important for the diagnosis of obesity (e.g., [22]) and other diseases such as hypertension [23–25] and diabetes [26, 27]. On the other hand, it is also important to separate the effects of age, gender, and weight from other risk factors such as blood pressure and blood glucose levels. By predicting these characteristics of a patient based on tongue images, we aimed to gain insights into how to capitalize on readily available tongue images to assess patients' health conditions noninvasively, conveniently, and inexpensively. The success of this proof-of-concept study will lay the important foundation for the development of a digital pipeline for the diagnosis of more important health conditions and diseases such as diabetes and hypertension.

We tested the general hypothesis that advanced machine learning techniques can be used to automatically extract crucial information from the color photographs of the tongue to accurately predict a patient's age, gender, and weight. This hypothesis is based on extensive research that has revealed the information about a patient's age, gender, and weight to be readily extracted from images of the patient's face, body, and MRI scans (e.g., [28–34]), thus suggesting that similar results can be obtained from tongue images.

However, direct research on whether the tongue can be used to index one's age, gender, and weight is very limited. Hsu et al. [35] and Jiang et al. [36] are likely among the few research groups that have investigated this question. They found that certain tongue characteristics were significantly associated with age and gender. For example, based on the statistical analysis of Hsu et al. [35], males have more red tongue color, more red dots, and yellower fur than females. Along with aging, more fissures, fewer teeth marks, and fewer red dots are evident in both male and female tongues. Both studies did not use tongue images to predict weight. However, their analyses have several limitations. First, Hsu et al. [35] used human-engineered tongue features rather than automatically learned features, which is time-consuming and may not capture all the information available on the tongue to differentiate gender, age, and weight. Second, both Hsu et al. [35] and Jiang et al. [36] used basic descriptive statistical analysis to compare different gender and age groups, rather than a comprehensive predictive analysis that takes all the features into account for age and gender predictions. These limitations may have impacted the reliability of their analysis and conclusions. Thus, to reliably and accurately predict patients' age, gender, and weight based on the tongue images, one needs to use more advanced computational techniques to gain a better understanding of the potential of the tongue as a tool for predicting age, gender, and weight, which was the primary goal of our study.

In the present study, we recruited a very large sample of participants that represented a wide range of ages, genders, and weights. We collected high-resolution color tongue images from 10,590 participants, with 5465 females. The tongue image of each participant was taken using iPhone cameras, and individual information, such as gender, age, and weight, was also recorded. To standardize the tongue images, we segmented tongue bodies from the original facial images using one of the state-of-the-art image segmentation techniques (i.e., Mask R-CNN [regional convolutional neural network]; He et al. [37]).

Second, we used a convolutional neural network (CNN) approach to automatically extract image features that might accurately predict the age, gender, and weight of patients. The CNNs employ a multilayer structure to analyze and automatically extract features from large datasets, which has been shown to be extremely successful for computer vision tasks (e.g., image processing: Krizhevsky, Sutskever, and Hinton [38]; facial recognition: Taigman et al. [39]). This technique is well suited for analyzing our tongue images because of the complex patterns and structures that can be found on the tongue. It is also less time-consuming than human-engineered features and avoids the need for subjective judgments by researchers. In other words, the CNNs can automatically and reliably extract potentially important features from tongue images that are related to the patient's age, gender, and weight.

Third, we also used deep CNNs to train and test computational models to predict patients' age, gender, and weight, separately. Since the last decade, deep CNNs, such as AlexNet [38], Inception [40], VGG [41], ResNet [42], ResNeXt [43], ShuffleNet [44], MobileNet [45], RegNet [46], Vision Transformer (ViT) [47], and EfficientNet [48] have been successfully used for computer vision tasks. Their application for face recognition has been highly successful in accurately determining an individual's age, gender, and weight based on facial features extracted by deep CNNs from their faces [49–57]. In this study, we used tongue images rather than facial images to predict an individual's age, gender, and weight. For evaluation, we use a fivefold cross-validation process. We collected one tongue image per participant, so our results are based on an interparticipant validation scheme.

We tested several specific hypotheses. First, we hypothesized that deep CNNs would be able to predict an individual's gender, age, and weight with an accuracy significantly above the chance level. Second, we hypothesized that the accuracies for the different measurements would be significantly different from each other, with gender models being the most accurate and weight models being the least. This hypothesis was based on the fact that gender and age are traditionally more visible and easier to extract from visual images than weight.

## 2. Method

### 2.1. Tongue Data

We recruited 10,590 participants, with 5465 females. They were ethnic Chinese and generally healthy. They were recruited from a Health Management Center where they underwent annual physical examinations. We collected the tongue image of each participant by using iPhones 7 and 8. The distance between the participants and the iPhone camera was about 50 cm. We also recorded individual information, including gender, age, and weight, as shown in Table 1. In our study, each tongue image corresponds to an individual, with associated information such as gender, age, and weight.

Our primary objective in this study was to predict age, gender, and weight using a conventional smartphone, which has not been extensively explored in previous research. The reason we focused on using a smartphone for data collection is to ensure a practical and feasible approach for noninvasive and remote disease diagnosis. While professional tongue image collection devices, such as tongue coating apparatus, might offer higher resolution images, they can be expensive and not as readily accessible as smartphones. By utilizing smartphones for data collection, we aimed to make the prediction process more affordable and widely applicable, potentially leading to inexpensive patient health monitoring and diagnosis.

### 2.2. Data Processing

**Figure 1:**
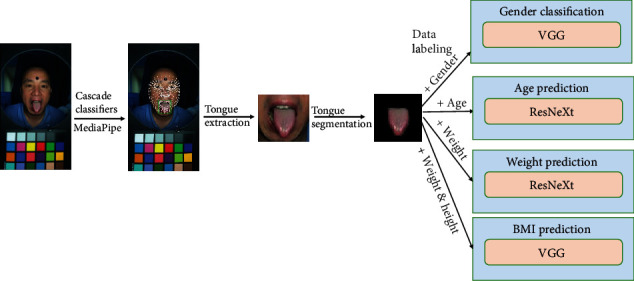
Data processing pipeline for tongue images.

For model predictions, including gender classification, age prediction, and weight estimation, we first undertake data processing steps as illustrated in Figure 1. To obtain the tongue image of each participant, we first applied color correction using a while balancing method to tongue images. Then, we used a Haar feature–based cascade classifier in combination with the MediaPipe library from OpenCV to segment the tongue region from the individual image. After that, we employed Mask R-CNN ([37]) to select the tongue body by masking the area of lips and chins. Note that the tongue image here refers to the image of the tongue's dorsal surface.

To create a tongue image dataset for prediction tasks, we labelled each tongue segment image based on its corresponding information (age, gender, and weight). For each prediction task (such as age prediction), we employed a fivefold cross-validation approach. This approach involved dividing the dataset into five distinct folds and trained and tested the model five times, each time using a different fold as the test set and the remaining four folds combined as the training set. To further ensure the robustness of our model, we repeated this entire fivefold cross-validation process three times. As a result, the model underwent a total of 15 testing iterations.

As shown in Figure 1, for gender classification, we utilize a pretrained VGG model (pretrained on ImageNet-1k). As it is a pretrained deep learning model, fine-tuning is necessary. Specifically, the VGG model, initially trained on a large public image dataset (i.e., the ImageNet dataset), is further trained using our tongue images. After training, we input the test tongue images into the model, which then generates the corresponding labels (i.e., female or male).

For age and weight prediction, we follow the same training and testing procedures with the respective image-label pairs (i.e., tongue image-age pairs or tongue image-weight pairs). However, we opt for the pretrained ResNeXt model instead of VGG to achieve higher accuracy.

### 2.3. Deep CNN Models

We used transfer learning for all our experiments to improve the performance of machine learning models and reduce the models' learning time. Specifically, we used fine-tuning technique, which is one of the three types of transfer learning methods. In fine-tuning, we retrained the whole pretrained model and updated all of the model's parameters for tongue feature-based classification or prediction tasks.

#### 2.3.1. VGG Architecture

**Figure 2:**
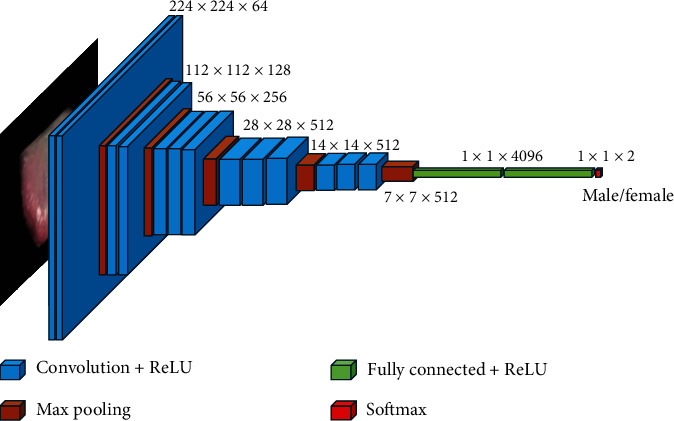
VGG architecture.

For the gender classification, we used VGG16 as the base architecture, which is pretrained on the ImageNet dataset. It is one of the most widely accepted architectures for facial image processing. The architecture of VGG16 is shown in Figure 2. Other popular pretrained models, such as ViT, EfficientNet, or InceptionV3, can also be used for our dataset. Our preliminary analyses showed that VGG16 achieved slightly better performance. We used a stochastic gradient descent (SGD) for optimization.

#### 2.3.2. ResNeXt Architecture

**Figure 3:**
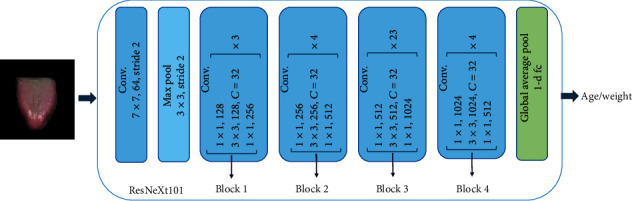
ResNeXt architecture.

To predict age and weight, we used ResNeXt-101 (for simplicity, we will refer to it as ResNeXt in the following section) as the base architecture built on top of ResNet. ResNeXt is constructed by repeating a building block that aggregates a set of transformations with the same topology (see Figure 3). Compared to a residual block, the ResNeXt block exposes a new dimension, cardinality *C* (the size of the set of transformations), as an essential factor in addition to depth and width. In this case, the *C* = 32. The main hyperparameters for fine-tuning the pretrained VGG and ResNet are listed in Table 2.

#### 2.3.3. Mask R-CNN Algorithm

**Figure 4:**
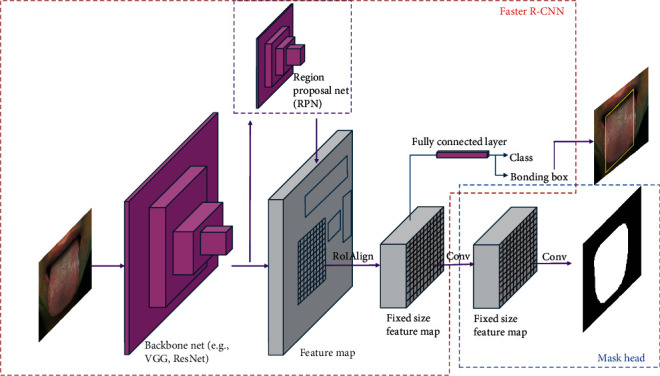
Mask R-CNN architecture.

We used the Mask R-CNN model pretrained on the Microsoft COCO (Common Objects in Context) dataset [58] to perform tongue segmentation. Mask R-CNN is an extension of Faster R-CNN [59] by adding a new branch for predicting an object (region of interest [RoI]) mask in parallel with the existing branch for classification and bounding box recognition. For our data, the RoI refers to the region of the tongue body. As shown in Figure 4, feature maps of an input image are abstracted by a backbone net (e.g., VGG or ResNet). These feature maps are then used by the region proposal network (RPN), a lightweight neural network, to generate multiple candidate RoIs where there might be an object. After that, a small feature map from each RoI is extracted and resized to the proper size. In this process, the alignment of the extracted features with the input image is achieved by the RoIAlign technique, which uses bilinear interpolation to compute the exact values of the features at four regularly sampled locations in each RoI bin. The values are then aggregated (using max or average). In the last step, the feature maps with a fixed size are used by fully connected layers to produce object classes and a bounding box. Fully connected layers also utilize feature maps to generate an object mask. After the object mask is generated, we can use pixel-wise operation between the input image and object mask to obtain the segment.

#### 2.3.4. Tongue Segmentation

To segment tongue bodies using Mask R-CNN, we randomly selected 1000 tongue region images as the training set. Initially, we manually annotated the training images using the VGG Image Annotator (VIA) [60] to produce masks of the tongue images, as illustrated in Figure 4. These training images, along with their corresponding masks, bounding boxes, and two class labels (i.e., background and tongue object), were then used to fine-tune the pretrained Mask R-CNN, which was initially trained on the COCO dataset. The parameter settings for fine-tuning Mask R-CNN are provided in Table 3. We used the SGD optimizer with a *learning rate* of 0.001, a *momentum* of 0.9, and a *weight decay* of 0.0001. The model underwent training for 90 epochs with 100 iterations per epoch while maintaining a batch size of 2, identical to the pretraining process. Mask R-CNN uses four main losses during training: RPN loss for accurate bounding box proposals, classification loss for correct object class identification, bounding box regression loss for precise bounding box adjustments, and mask loss for accurate object shape representation. These losses are backpropagated to update the model's parameters and improve prediction accuracy. The source code of Mask R-CNN is available at https://github.com/facebookresearch/Detectron.

**Figure 5:**
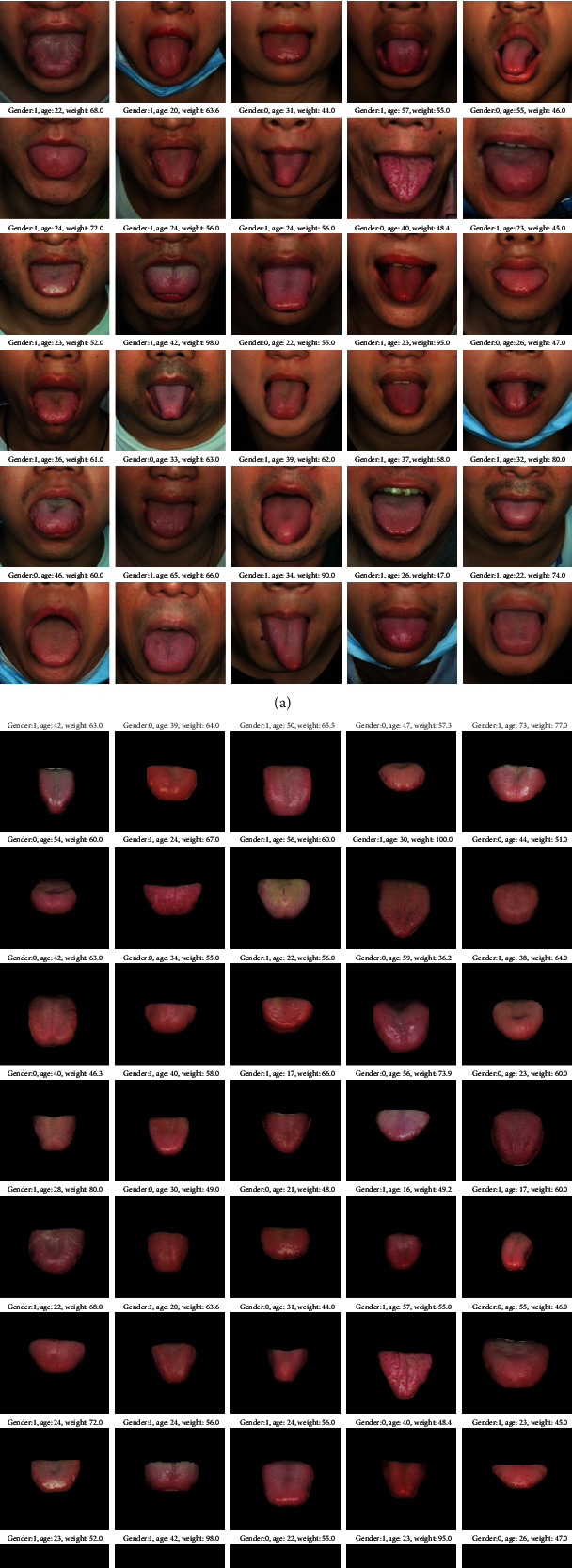
Examples of tongue segments. (a) Tongue regions extracted from the original images using OpenCV. (b) Segmented tongue bodies using Mask R-CNN.

During the testing process, we used the fine-tuned Mask R-CNN to automatically generate tongue bodies for the remaining 9590 tongue images (out of a total of 10590 images). It is important to note that the accuracy of Mask R-CNN relies on the number of manually crafted training masks. In our study, with 1000 training masks, we achieved a 100% correct segmentation of the remaining 9590 tongue bodies from their respective tongue images.  Figure 5 illustrates some examples of segmented tongue bodies obtained during the testing process.  Figure 5(a) showcases the tongue regions extracted from the original images using OpenCV (i.e., the input images for Mask R-CNN). On the other hand, Figure 5(b) presents the corresponding segmented tongue bodies, which are the output of Mask R-CNN after the automated segmentation process.

## 3. Results

### 3.1. Gender Classification

Gender classification was accomplished by first collecting a dataset of tongue images along with corresponding gender labels. Each tongue image was associated with a participant's gender. The tongue images underwent preprocessing steps, such as tongue region extraction, tongue body segmentation, and data labelling, to prepare them for training (as shown in Figure 1). The pretrained VGG16 model was fine-tuned on the tongue image dataset, adjusting its parameters to effectively classify the gender based on tongue features. After training, the model is capable of classifying the gender of new tongue images as either male or female.

All the gender classification results are measured by classification accuracy, sensitivity (or *true positive rate*), specificity (or *true negative rate*), and AUC (area under the receiver operating characteristic [ROC] curve). AUC measures the entire two-dimensional area underneath the entire ROC curve. A ROC curve is a graph showing the performance of a VGG model at all classification thresholds. This curve plots two parameters: *true positive rate* and *false positive rate*. In the case of gender classification, the *true positive rate* represents the ratio of correct classification of male tongue images to the total male tongue images; the *false positive rate* means the ratio of incorrect classification of female tongue images to the total female tongue images, and the *true negative rate* represents the ratio of correct classification of female tongue images to the total female tongue images (i.e., 1*—false positive rate*).

We used fivefold cross-validation to evaluate the performance of pretrained VGG fine-tuned on the RGB tongue images. The experiments are repeated three times. The gender classification accuracies and the AUCs of pretrained VGG are shown in Table 4. Transfer learning with VGG using RGB tongue images consistently achieved impressive results for all cross-validations, with overall means of accuracy being 79.5% and AUC at 88.2% and overall standard deviations at 0.8% and 0.6%, respectively.

In this study, we compared a pretrained VGG model with two state-of-the-art deep learning models, namely, the ViT and EfficientNet, as well as two statistical methods, support vector machine (SVM) and XGBoost, to evaluate their performance in gender classification. The results of this comparison are shown in Table 5. Both ViT (pretrained on ImageNet-21k and fine-tuned on ImageNet-1k) and EfficientNet (pretrained on ImageNet-1k) are pretrained models. For SVM and XGBoost, we used two approaches to train and test the models. In the first approach, whole images were directly fed into the model. In the second approach, image features were extracted first and then used as inputs. We used two types of features: color features and texture features. Color features were extracted using color histograms, while texture features were based on the gray-level cooccurrence matrix (GLCM) [61]. The color features and texture features were concatenated to form image features.

Based on the comparison results, we can conclude that deep learning-based methods significantly outperformed statistical methods for the gender prediction task by improving the performance of the best statistical method (XGBoost) by around 6.5% in accuracy. Moreover, VGG performed better than other more complex models, namely, ViT (78.4%) and EfficientNet (76.9%).

### 3.2. Age Prediction

For age prediction, we used the same dataset of tongue images with corresponding age-in-year labels. Similar to the gender classification task, the tongue images were preprocessed to ensure they were ready for training. We then used the ResNeXt architecture (as shown in Figure 3). The pretrained ResNeXt model was fine-tuned on the tongue image dataset, adjusting its parameters using regression to predict the age based on tongue features. Following the training phase, the model was capable of predicting the age of new, unseen tongue images. It outputs a numerical value representing the predicted age in years. To evaluate the model's performance, a test set was used, and the mean absolute error (MAE) and correlation coefficient were used as metrics. The MAE measures the average absolute difference between the predicted age and the true age, while the correlation coefficient measures the linear relationship between the predicted and true ages.

**Figure 6:**
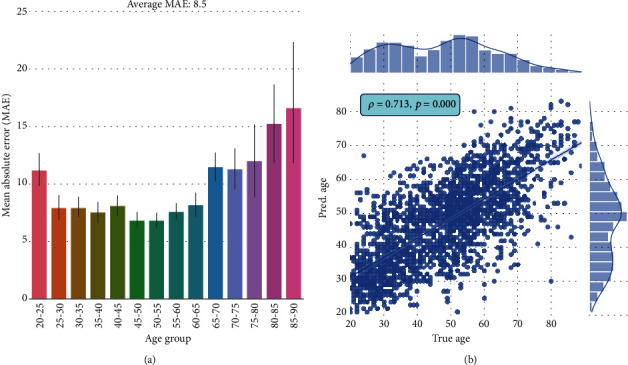
Age (years) prediction of pretrained ResNeXt fine-tuned on RGB tongue images. (a) Mean absolute errors (MAEs) between the predicted and true ages for 14 age groups. (b) Correlation between the predicted ages and the true ages.

**Figure 7:**
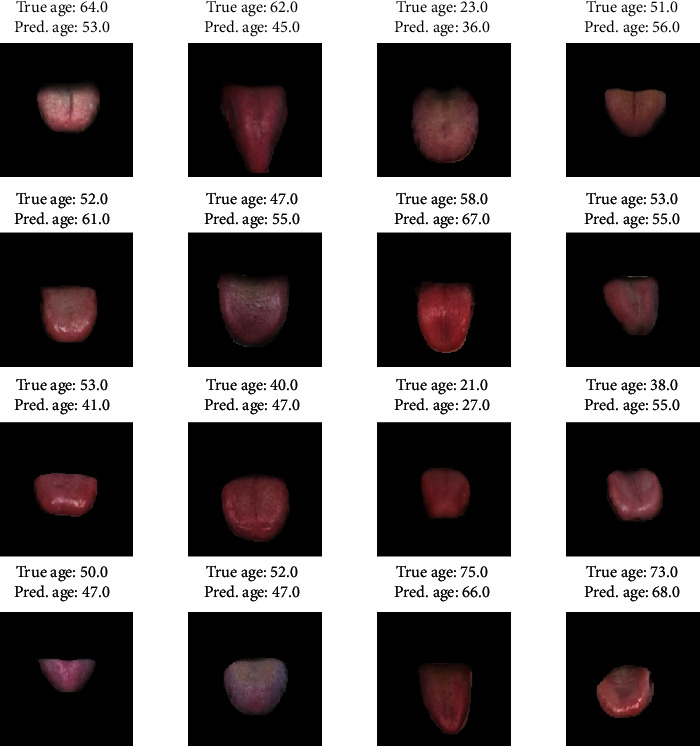
Examples of predicted ages of pretrained ResNeXt fine-tuned on RGB tongue images.

The prediction results of ResNeXt trained on RGB tongue images are shown in Figure 6.  Figure 6(a) illustrates the MAEs between the predicted and true ages for 14 age groups. The predicted results for groups aged 25–65 are quite good, with mean MAEs below 8 years. Although the mean MAEs of age groups 20~25, 65~70, 70~75, and 75~80 are relatively high, they are not bigger than 12.5 years. Since the number of participants older than 80 is fairly small (as shown in Figure 6(b)), not surprisingly, the prediction results are not satisfied with MAEs above 15 years. A moderately strong correlation between the predicted ages and the true ages is shown in Figure 6(b), with a correlation coefficient *ρ* = 0.71.  Figure 7 shows some examples of predicted ages from the test set.

To evaluate the performance of age prediction, we compared a pretrained ResNeXt model with other models, including two advanced deep learning methods, the ViT and ResNet-50 (pretrained on ImageNet-1k), as well as two statistical methods, Lasso and random forest. The evaluation results are shown in Table 6. Similar to the evaluation of gender classification, we used two types of input data for the statistical methods: whole images and extracted image features.

The comparison results for age prediction show a similar conclusion to those for gender classification: deep learning-based methods significantly outperformed statistical methods by reducing the MAE of random forest by around 3 years and increasing the correlation coefficient (*ρ*) by approximately 0.3. Among these three deep learning methods, ResNeXt achieved the best performance.

### 3.3. Weight Prediction

We used the same dataset of tongue images with corresponding weight labels. As with the other two tasks, the tongue images were preprocessed to ensure they were appropriately prepared for training. The ResNeXt architecture was chosen for this task as well, and the pretrained ResNeXt model was fine-tuned on the tongue image dataset, using regression to predict the weight based on tongue features. After the training phase, the model was capable of predicting the weight of new tongue images. It outputs a numerical value representing the predicted weight.

**Figure 8:**
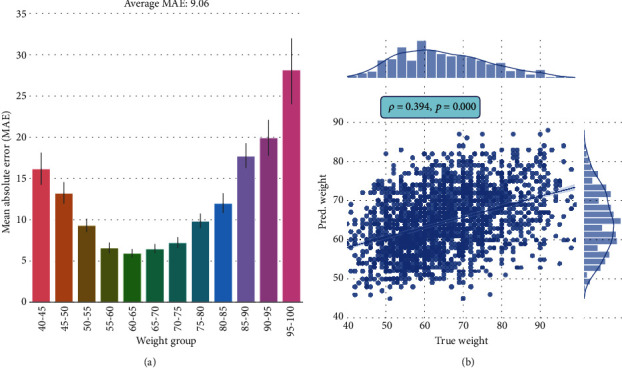
Weight (kilograms) prediction of pretrained ResNeXt fine-tuned on RGB tongue images. (a) MAEs between the predicted and true weights for 12 weight groups. (b) Correlation between the predicted weights and the true weights.

The results of weight prediction are shown in Figure 8. As shown in Figure 8(b), the correlation coefficient between predicted weights and true weights is 0.394, which is statistically significant above the chance level. However, the MAEs are rather large. Except for middleweight groups, 50~55 kg, 55~60 kg, 60~65 kg, 65~70 kg, 70~75 kg, and 75~80 kg, the mean MAEs of other groups are above 10 kg.

Similar to the age prediction task, we compared ResNeXt with two other pretrained deep learning models, namely, ViT and ResNet-50, as well as with Lasso and random forest. The comparison results are presented in Table 7. Unlike the conclusions drawn from gender and age predictions, statistical models generally achieve higher correlation coefficients (*ρ*) between predictions and ground truth than deep learning models; however, their MAEs are also larger. For instance, random forest achieves a slightly higher *ρ* value (0.40) compared to ResNeXt (0.39), but it also has a significantly larger MAE (11.63 compared to 9.06 for ResNeXt). Therefore, using the MAE as the main criterion, we can conclude that ResNeXt generally outperformed both the statistical and other deep learning methods.

## 4. Discussion

In this proof-of-concept study, we explored whether advanced machine learning techniques can be used to reveal patient information based on tongue images alone. Using pretrained deep CNN models and a large dataset of tongue images, we confirmed the hypothesis that human tongue images contain sufficient information about a patient's age, gender, and weight, and thus, CNN-based methods are able to extract such information to make reliable classifications or predictions with accuracy significantly above the chance level. This finding suggests that our methodology is a potentially highly viable approach to developing a digital pipeline to use tongue images for patient health monitoring and disease diagnosis purposes.

The success of our deep CNN-based age prediction models confirms the existing anatomical and physiological evidence that with increased age, our tongue indeed undergoes systematic aging [35, 36, 62–66]. To the best of our knowledge, we are the first to demonstrate that deep CNN models are able to make age predictions based on the tongue image alone. While some studies, such as Hsu et al. [35] and Jiang et al. [36], have explored the correlations of gender and age factors with tongue images, their approach primarily relied on basic descriptive statistical analysis to compare different gender and age groups. However, these studies did not conduct a comprehensive predictive analysis that takes all the features into account for accurate age and gender predictions based on tongue images. The reason for our success could be due to the larger number of tongue images we collected, which enabled us to train more complex computational models, and the use of the deep CNN approach that does not need to preprocess all the raw tongue images but automatically capture information from all the raw pixels to extract useful age-related information.

Our proposed models' gender classification achieves high accuracy, with the best results showing a mean accuracy of 79.5% ± 0.8%. The high accuracy of gender classification may be due to the fact that male and female tongues are distinct in terms of tongue morphology and hemoglobin concentrations [35, 36, 67]. The distinct myoglobin and hemoglobin levels between males and females [68, 69] might result in significant color differences between male tongues and female tongues. Our results confirm the hypothesis that there are disparities between male tongue features and female tongue features.

The results of weight prediction were not as good as that of age and gender. The correlation coefficient between predicted weights and true weights was 0.39, and the MAEs of most weight groups were above 10 kg. Nashi et al. [70] reported that patient body weight is significantly associated with a larger proportion of fat within the tongue. However, Kim et al. [9] suggested that most tongue fat is deposited under the tongue surface, especially in the back of the tongue. Because we trained our models on the dorsal surface images of the tongue, we might have missed the most crucial information to predict the patient's weight. As a result, our CNN models failed to make a weight prediction with an accuracy as high as that of age and gender.

Nevertheless, the present study provided a proof-of-concept prototype by using pretrained deep CNN models to automatically extract task-specific features from the tongue images to train computational models to predict patients' age and gender with high accuracy and weights with moderate accuracy. The present findings provide strong evidence to suggest the feasibility of using the same approach and digital pipeline to train computational models to predict other medical conditions or diseases such as diabetes, hypertension, and even influenza infections. In the future, once a large image set of tongues is labelled with the ground truths of a medical condition (e.g., the presence or absence of diabetes), our approach and pipeline can be leveraged to train and test computational models for making predictions about the likelihood of a patient having such a condition. The models built upon our approach hold the potential to be implemented on smartphones or websites, providing patients and physicians with convenient access to the predictive capabilities, thus aiding in medical diagnosis or patient disease monitoring.

We emphasize that the reason we focused on using smartphones for data collection was to ensure a practical and feasible approach for noninvasive and remote disease diagnosis. While professional tongue collection devices, such as tongue coating apparatus, may offer higher resolution images, they often come with higher costs and might not be as readily accessible as smartphones. By leveraging smartphone technology, we aimed to create a more accessible and affordable method for tongue image data collection, enabling widespread adoption and making noninvasive medical prediction and diagnosis more accessible to a broader population.

### 4.1. Limitations and Future Studies

The present research has several limitations that need to be addressed in future studies. First, by capturing a full face image and cropping the tongue area, we did not capture the finer details of the tongue surface. Had we focused exclusively on the tongue within the full frame of the camera, we would have achieved a higher quality tongue image. Further, the tongue image data used in this study was collected using iPhone 7 and 8 devices in 2021 in China, which might result in lower quality compared to images captured with more modern smartphones. With higher-quality tongue images, the proposed method could likely achieve even higher accuracy for age, gender, and weight predictions. Future research could either continue to use smartphones but with increased image resolution or use specialized optical devices to obtain higher-quality tongue images.

Second, although we obtained a large set of tongue images from a large sample of patients, the age and weight distributions were not even, which led to high inaccuracies for certain age groups or weight groups. Future studies should ensure the acquisition of tongue images from patients that are evenly distributed in terms of age and weight.

Third, the tongue images are all from the frontal perspective. Additional images of the same tongues from the ventral perspective in future studies will likely improve our accuracy. This is because much information about age, gender, and weight can be gleaned from this perspective. For example, it is well established that there are significantly more fat deposits under the tongue than on the tongue, and thus with ventral tongue images, at least the weight prediction will likely improve. Also, the vasculature is much richer in the ventral surface of the tongue than the dorsal one [71, 72], which should provide additional information about age- or gender-related changes in hemoglobin concentrations. Thus, in the future, we might combine both dorsal surface and ventral surface tongue images to achieve more accurate prediction results.

Fourth, to build a massive dataset, we collected data from individuals undergoing annual physical examinations at a Health Management Center in China. Therefore, the majority of data samples were from healthy individuals, which could limit the generalizability of our findings. Based on the fivefold cross-validation results, the model performs well on the unseen subgroup (or fold) of the current homogeneous dataset. However, there could be overfitting when predicting unseen data from outside the ethnic Chinese group or from people with different health conditions. Therefore, in future work, we will collect more diverse datasets from people of different ethnic groups and health conditions to train the model and improve its generalizability, thereby avoiding the overfitting problem.

Fifth, our current tongue image dataset consists only of participants in healthy conditions. As a result, we are unable to classify a white greasy tongue as either normal or exhibiting a specific coating due to the absence of labelled images depicting greasy coating and rotten coating tongues in our dataset. Future research could address this issue by using labelled tongue image datasets. With such datasets, we could include white greasy coating tongue images from unhealthy patients to investigate whether these images would affect the predictive accuracies of gender, age, and weight.

Lastly, although our study focuses on using tongue images to infer gender, age, and weight, the findings of this study demonstrate the viability of applying advanced machine learning approaches to tongue images to predict other patient conditions. For example, Shi et al. [73] provided an annotated dataset of tongue images supporting geriatric disease diagnosis. Although their approach is qualitative and emphasizes visual examination and diagnostic interpretation directly from images, their results illustrate the possibility of using the same advanced machine learning approach as those used in the present study to reveal patient health and disease conditions solely based on tongue images. This possibility should be explored in future research.

## 5. Conclusion

The present study introduced a novel approach to predict patient age, gender, and weight inferences based on tongue images using pretrained deep CNNs. Our results demonstrated that the deep CNN models trained on dorsal tongue images produced excellent results for age prediction with a Pearson correlation coefficient of 0.71 and a MAE of 8.5 years. We also obtained an excellent classification of gender, with a mean accuracy of 79.5% and an AUC of 88.2%. The model also obtained a moderate level of accuracy for weight prediction, with a Pearson correlation coefficient of 0.39 and a MAE of 9.06 kg. These findings support our hypothesis that the human tongue contains crucial information about a patient.

This study demonstrated the feasibility of using the pretrained deep CNNs along with a large tongue image dataset to develop computational models to predict patient medical conditions for noninvasive, convenient, and inexpensive patient health monitoring and diagnosis. However, since most of the data samples were collected from healthy individuals, the generalizability of our findings may be limited. While the fivefold cross-validation results indicate that the model performs well on unseen subgroups (or folds) within the current homogeneous dataset, there is a potential for overfitting when predicting data from outside the ethnic Chinese group or from individuals with different health conditions. To address this, future work will involve collecting more diverse datasets from various ethnic groups and health conditions to train the model, thereby enhancing its generalizability and mitigating the risk of overfitting.

## Figures and Tables

**Table 1:** Means, standard deviations, and min and max values of participants' age and weight.

**Features**	**Min**	**Max**	**Mean**	**Std.**
Age	20	89	48	15.5
Weight (kg)	40	99.5	65.4	11.8

**Table 2:** Main hyperparameter values for fine-tuning pretrained VGG and pretrained ResNet models.

**Models**	**Hyperparameters**	**Gender**	**Age**	**Weight**
Pretrained VGG (optimizer: SGD)	Batch size	32	—	—
	Epochs	50	—	—
	Learning rate	0.0001	—	—
	Momentum	0.9	—	—

Pretrained ResNeXt (optimizer: Adam)	Batch size	—	128	32
	Epochs	—	90	140
	Initial learning rate	—	0.001	0.001
	Gamma	—	0.95	0.97

*Note:* For fine-tuning the pretrained VGG, we used SGD optimizer and fixed the learning rate = 0.0001. For fine-tuning the pretrained ResNeXt, we used Adam optimizer with initial learning rate being 0.001 for both age and weight predictions and exponential learning rate decay with a multiplicative factor of learning rate decay (i.e., gamma) being 0.95 and 0.97 for age and weight predictions, respectively.

**Table 3:** The main hyperparameters for fine-tuning the pretrained Mask R-CNN.

**Model**	**Hyperparameters**				
	**Epochs**	**Step per epoch**	**Learning rate**	**Momentum**	**Weight decay**
Mask R-CNN	30	100	0.001	0.9	0.0001

*Note:* When fine-tuning the pretrained Mask R-CNN, we used SGD optimizer and fixed the learning rate, by setting learning rate = 0.001, momentum = 0.9, and weight decay = 0.0001.

**Table 4:** Gender classification accuracy (percent) and AUC (percent) of pretrained VGG fine-tuned on RGB tongue images.

		**Fold 1**	**Fold 2**	**Fold 3**	**Fold 4**	**Fold 5**	**Mean (± std.)**
Repeat 1	Accuracy (%)	80.0	78.7	80.3	79.4	79.0	79.5 (± 0.6)
	AUC (%)	88.5	87.6	89.0	87.6	88.1	88.2 (± 0.6)
	Sensitivity (%)	78.8	77.3	81.5	81.5	84.3	80.7 (± 2.4)
	Specificity (%)	81.2	80.1	79.2	77.3	74.5	78.5 (± 2.4)

Repeat 2	Accuracy (%)	79.4	79.4	78.6	79.9	78.9	79.2 (± 0.4)
	AUC (%)	88.6	88.3	87.7	88.9	87.1	88.1 (± 0.7)
	Sensitivity (%)	78.9	71.2	76.8	79.7	73.8	76.1 (± 3.2)
	Specificity (%)	79.8	87.1	80.4	80.0	83.1	82.1 (± 2.8)

Repeat 3	Accuracy (%)	79.9	79.7	81.9	78.4	79.4	79.9 (± 1.1)
	AUC (%)	88.0	88.7	88.9	87.8	88.2	88.3 (± 0.4)
	Sensitivity (%)	77.9	83.3	80.4	80.7	77.8	80.0 (± 2.0)
	Specificity (%)	81.7	76.6	83.2	76.3	81.1	79.8 (± 2.8)

Mean (± std.)	Accuracy (%)	—	—	—	—	—	79.5 (± 0.8)
	AUC (%)	—	—	—	—	—	88.2 (± 0.6)
	Sensitivity (%)	—	—	—	—	—	78.9 (± 3.3)
	Specificity (%)	—	—	—	—	—	80.1 (± 3.1)

Abbreviation: std. = standard deviation.

**Table 5:** Comparison results of gender classification.

**Method**	**Accuracy (%)**					
	**Fold 1**	**Fold 2**	**Fold 3**	**Fold 4**	**Fold 5**	**Mean (± std.)**
ViT	80.2	78.3	76.3	78.3	79.1	78.4 (± 1.3)
EfficientNet	77.5	76.0	78.6	75.5	76.7	76.9 (± 1.1)
SVM (image)	70.0	72.2	71.1	70.0	68.7	70.4 (± 1.2)
SVM (feat.)	67.2	67.7	67.8	67.0	65.9	67.1 (± 0.6)
XGBoost (image)	71.2	72.6	70.6	70.4	70.4	71.0 (± 0.8)
XGBoost (feat.)	73.3	72.4	74.2	71.5	72.9	72.9 (± 0.9)
VGG	80.0	78.7	80.3	79.4	79.0	79.5 (± 0.6)

*Note:* The pretrained VGG is compared with two state-of-the-art deep learning models, including Vision Transformer (ViT) and EfficientNet, and two statistical methods, including SVM and XGBoost.

**Table 6:** Results of the age prediction comparison.

**Method**	**MAE**	**Correlation coefficient (** **ρ** **)**
ViT	10.7	0.50
ResNet-50	9.0	0.67
Lasso (image)	12.0	0.36
Lasso (feat.)	11.7	0.41
Random forest (image)	11.5	0.44
Random forest (feat.)	11.8	0.41
ResNeXt	8.5	0.71

*Note:* We compared a pretrained ResNeXt model with other state-of-the-art deep learning pretrained models, including ViT and ResNet-50, as well as two statistical methods, Lasso and random forest.

**Table 7:** Results of the weight prediction comparison.

**Method**	**MAE**	**Correlation coefficient (** **ρ** **)**
ViT	10.61	0.26
ResNet-50	9.34	0.32
Lasso (image)	12.10	0.33
Lasso (feat.)	11.84	0.37
Random forest (image)	11.63	0.40
Random forest (feat.)	11.77	0.38
ResNeXt	9.06	0.39

*Note:* A pretrained ResNeXt model was compared with other pretrained models, including ViT and ResNet-50, as well as two statistical methods, Lasso and random forest.

## Data Availability

Data request should be sent to lislucy@163.com.
